# Cytokine Profile in Women Affected by Chikungunya: Analysis of a Prospective Cohort in Northeastern Brazil

**DOI:** 10.1590/0037-8682-0317-2025

**Published:** 2025-12-15

**Authors:** Thalita do Nascimento Silva, Alberto Rubens Siqueira Nogueira Leal, Francisca Kalline de Almeida Barreto, Luís Arthur Brasil Gadelha Farias, Livia Mendes de Almeida, Rafhaella Nogueira Della Guardia Gondim, Marco Antônio de Freitas Clementino, Edson Holanda Teixeira, Moacyr Jesus Barreto de Melo Rêgo, André Siqueira, Juliana Navarro Ueda Yaochite, Luciano Pamplona de Góes Cavalcanti

**Affiliations:** 1 Centro Universitário Christus, Faculdade de Medicina, Fortaleza, CE, Brasil.; 2 Universidade Federal do Ceará, Programa de Pós-Graduação em Patologia, Fortaleza, CE, Brasil.; 3 Universidade Federal do Ceará, Programa de Pós-Graduação em Microbiologia, Fortaleza, CE, Brasil.; 4 Universidade Federal do Ceará, Programa de Pós-Graduação em Saúde Pública, Fortaleza, CE, Brasil.; 5 Universidade Federal do Ceará, Programa de Pós-Graduação em Fisiologia e Farmacologia, Fortaleza, CE, Brasil.; 6 Universidade Federal de Pernambuco, Departamento de Farmácia, Recife, PE, Brasil.; 7 Fundação Oswaldo Cruz, Rio de Janeiro, RJ, Brasil.

**Keywords:** Chikungunya Fever, Cytokines, Inflammation, Chikungunya virus

## Abstract

**Background:**

Chikungunya (CHIK) is an acute febrile arthritic illness caused by *Aedes aegypti* and *Aedes albopictus* mosquitoes. Understanding the pathophysiology of CHIK is crucial because of the wide distribution of cases and the lack of specific treatments or validated biomarkers. This study aimed to describe the cytokine profiles of female patients with laboratory-confirmed CHIK across the three clinical phases of the disease, monitored at an outpatient clinic in Northeastern Brazil. Additionally, this study evaluated whether cytokine levels were associated with persistent arthralgia and the presence of comorbidities.

**Methods:**

This was a prospective cohort study conducted from 2021 to 2024, including 40 female patients and 10 heathy controls (women aged ≥18 years, without comorbidities, not taking medications and non-reactive serologies for CHIK). Blood samples were collected at five time points (0, 21, 90, 180, and 360 days after the symptom onset).

**Results:**

More than half of the patients reported persistent pain. Among CHIK-infected women, interleukin (IL)-10 levels remained elevated from day 21, with statistically significant differences between D0 and D180 and D0 and D360 (p = 0.027). IL-18 levels increased significantly between D0 and D21 (p = 0.020).

**Conclusions:**

Dynamic cytokine behavior throughout the CHIK phase has been described in other studies and may be influenced by host immunogenetics and other factors.

## INTRODUCTION

Chikungunya (CHIK) disease is a major public health concern worldwide. Between 2011 and 2020, more than 18 million cases were reported across 110 countries, with substantial economic impacts owing to direct and indirect costs^1^ ^,^ ^2^. In recent years, an increase in severe cases, chronic and deforming arthropathies, cognitive and physical sequelae, and excess mortality has been observed during the epidemic periods. These developments have raised concerns among health authorities, particularly regarding the global risk of infection. The widespread presence of *Aedes* vectors, favored by unplanned urbanization and climate change, further increases the potential for new outbreaks, especially in low- and middle-income countries^3^ ^-^ ^7^.

Although immune responses during CHIK infection have been increasingly described, the mechanisms underlying its pathogenesis remain poorly understood. Nevertheless, some studies have suggested that cytokines may serve as potential biomarkers associated with disease severity and chronicity and could become future therapeutic targets^8^ ^-^ ^10^. The beneficial role of cytokines is known, but their serum levels can fluctuate according to the phase of CHIK, such as interleukin (IL)-18, granulocyte macrophage colony stimulating fator (GM-CSF), and interferon-gamma (IFN- γ), which are present in high concentrations in the acute phase. IL-1B, IL-6, and monocyte chemoattractant protein-1(MCP-1) levels are associated with symptom severity. Other molecules such as IL-2, IL-4, IL-10, and IL-17A may be associated with unfavorable disease outcomes such as chronic and debilitating arthropathy^8^ ^,^ ^9^ ^,^ ^11^ ^-^ ^18^.

This study aimed to evaluate the behavior and concentrations of pro- and anti-inflammatory cytokines during the three clinical phases of the disease (acute phase, lasting up to 14 days after the first symptoms; post-acute phase, lasting from 15 days to 3 months; and chronic phase, in which joint symptoms persist for >3 months) in women enrolled in a cohort in Northeast Brazil. In addition, we investigated the relationship between cytokine levels and persistent joint pain (arthralgia reported by patients for ≥3 months) and the presence of comorbidities^19^.

## METHODS

### Study design and Ethical aspects

This prospective cohort study was conducted between 2021 and 2024 in northeastern Brazil. This study was part of the Clinical and Applied Research Network in Chikungunya (REPLICK), a prospective, multicenter, outpatient cohort study conducted in Brazil to investigate the natural history of CHIK. REPLICK participants were followed up longitudinally for 3 years, starting from the first symptoms. This study assessed musculoskeletal manifestations, chronic pain, quality of life and mental health in individuals infected with chikungunya virus (CHIKV)^20^. This study included female participants with laboratory-confirmed CHIKV infection and healthy controls. Blood samples were collected at multiple time points to assess the cytokine profiles throughout the acute and chronic phases of the disease. 

The study protocol was approved by the Brazilian National Research Ethics Commission (Comissão Nacional de Ética em Pesquisa-CONEP) under CAAE number 07936919.8.1001.5262 and Opinion Report number 3.352.657. 

All eligible participants received information about the study and signed an informed consent form before enrollment^20^. The study was conducted in accordance with the Declaration of Helsinki and Brazilian Guidelines for Research Involving Human Subjects. 

### Inclusion Criteria

Female patients with CHIK who were followed up at the REPLICK outpatient clinic in Ceará, Northeast Brazil, aged ≥18 years, and presented with a clinical picture suggestive of CHIK were included. Suspected cases were laboratory-confirmed through serology by detecting IgM antibodies (collected during the acute phase), IgG antibodies (a fourfold increase in anti-CHIKV-specific antibody titers in samples collected at least 2-3 weeks apart), and/or reverse transcription-polymerase chain reaction (RT-PCR). 

Only laboratory-confirmed cases were included. The analysis was limited to female participants, as they constituted the majority of the blood samples collected and registered in the REPLICK cohort. The number of male samples was insufficient for a meaningful comparison. Moreover, female patients demonstrated higher adherence to the five scheduled follow-up visits, which contributed to a reduced loss to follow-up and improved data consistency.

### Exclusion Criteria

Women with autoimmune inflammatory arthropathies, such as rheumatoid arthritis, systemic lupus erythematosus, and spondyloarthritis, as well as those with other acute febrile illnesses, such as dengue and Zika, confirmed using serology or RT-PCR, were excluded. 

### Blood Samples

Samples were collected at five time points following the medical consultation. The first was during the acute phase of the disease, no more than 3 days after the first symptoms of CHIK. The second sample was collected during the post-acute phase (21 d after symptom onset). The third, fourth, and fifth samples were obtained at distinct time points during the chronic phase (90, 180, and 360 days after symptom onset). A margin of ±3 days was considered for each sample collection. Samples from 50 women were included: 40 with laboratory-confirmed CHIKV infection and 10 healthy controls (women aged ≥18 years, without comorbidities, no medications, and non-reactive serologies of IgM and IgG for CHIK). Blood samples were collected from all the participants.

### Serum Cytokines

Cytokine profiling was performed using Milliplex® assays (Milliplex® Human Cytokine/Chemokine/Growth Factor Panel A Magnetic Bead Panel, Merck KGaA, Darmstadt, Germany) targeting 10 analytes (GM-CSF, IFN-γ, IL-1β, IL-2, IL-4, IL-6, IL-10, IL-17A, IL-18, and MCP-1), following the manufacturer's instructions^21^. Although other cytokines such as tumor necrosis factor-alpha (TNF-) participate in the pathophysiology of CHIK^9^, only the cytokines mentioned above were included, due to the availability of preformed cytokine kits by the manufacturer. 

### Statistical Analysis

Differences in serum cytokine levels between groups were compared using a non-parametric Kruskal-Wallis test, with statistical significance set at p < 0.05. Pearson’s correlation coefficient was calculated to assess the associations, classified as weak (0.1-0.3), moderate (0.4-0.6), strong (0.7-0.9), or perfect (1.0). Linear regression analysis was performed to identify the predictors of symptom resolution within one month in the study cohort.

Cytokine concentrations were analyzed using the Xponent® software and expressed in picograms per milliliter (pg/mL). The results were validated by comparison with the calibration standards and controls, according to the manufacturer's instructions^21^.

In addition to blood samples, clinical reports were used to collect sociodemographic and medical history data, including age, place of origin, comorbidities, medication use, and presence or persistence of arthralgia (joint pain for > 3 months).

Categorical variables are reported as counts and relative frequencies (percentages). Associations between categorical variables were assessed using the chi-square test or Fisher’s exact test, depending on the expected frequencies. Continuous variables were first assessed for normality using the Shapiro-Wilk test and graphical analysis with Q-Q plots, histograms, and quantitative measures of dispersion, skewness, and kurtosis. Variables with a normal distribution are expressed as mean ± standard deviation, whereas non-normally distributed variables are reported as medians and interquartile ranges. For comparisons between two independent groups, Student’s *t*-test or Mann-Whitney *U* test was applied, depending on the data distribution.

The Friedman test was used for comparisons involving multiple time points in dependent groups (paired data). When a significant overall difference was observed (p < 0.05), post hoc pairwise comparisons were performed using the paired Wilcoxon test with Holm correction to control for type I errors. The significant Friedman test results indicate a global trend of change over time. However, individual pairwise differences may not reach statistical significance after correction, reflecting a global effect without necessarily detectable, pointwise changes.

To visualize the dynamics of continuous variables (cytokines) over time, alluvial plots were constructed by categorizing cytokine levels into qualitative tiers (“low,” “medium,” and “high”). Categorization was based on tertiles: values below the 33rd percentile = “low,” between the 33rd and 66th percentiles = “medium,” and above the 66th percentile = “high.” The tertiles of the distribution of each cytokine were evaluated at different time points (D0, D21, D90, D180 and D360.). A p-value < 0.05 was considered statistically significant. All analyses were performed using R software (version 4.3.1; R Core Team, 2021), including *tidyverse*, *ggplot2*, *ggpubr*, *moments*, *rstatix*, *flextable*, and *ggalluvial*.

## RESULTS

### Patients’ Characteristics

The mean age of the women was 47.3 ± 16.4 years. Persistent pain was reported by more than half of the participants (57.9%), and comorbidities were present in 45.0%. Among them, systemic arterial hypertension (27.5%) and diabetes mellitus (17.5%) were the most prevalent. During the first consultation, 90% of the women reported using pain medications, most commonly dipyrone (62.5%), paracetamol (27.5%), and nonsteroidal anti-inflammatory drugs (22.5%). 

At the initial consultation during the early symptomatic phase (D0), women with CHIK showed significantly lower IL-18 levels than those in the control group [581.7 (225.0-5,010.6) pg/mL vs. 5,256.4 (5,003.5-5,728.1) pg/mL, p = 0.012] (Table 1).

**TABLE 1:** Cytokine levels at D0 in blood samples from patients with chikungunya and comparison with the control group

Cytokines	Control n = 10^1^	CHIKV n = 38^1^	p-value^2^
GM-CSF pg/mL (D0)	36,495.1 (20,001.3-40,000.0)	40,000.0 (20,001.3-40,000.0)	0.743
IFN-γ pg/mL (D0)	10,850.7 (10,001.1-20,000.0)	4,348.0 (1,088.0-11,116.9)	0.128
IL-1β pg/mL (D0)	18,877.8 (2.9-25,000.0)	9,695.2 (489.1-23,265.5)	0.792
IL-2 pg/mL (D0)	8,143.9 (5,000.5-15,062.0)	5,652.5 (5,000.1-10,000.0)	0.440
IL-4 pg/mL (D0)	7,822.1 (5,000.2-10,075.1)	5,302.9 (5,000.1-10,000.0)	0.508
IL-6 pg/mL (D0)	1,886.8 (1.4-7,325.8)	1,039.0 (11.0-2,377.2)	0.533
IL-10 pg/mL (D0)	20,004.6 (12,737.3-25,305.9)	6,774.3 (2,109.5-20,011.4)	0.128
IL-17A pg/mL (D0)	11,884.0 (10,003.2-19,357.7)	10,001.5 (3,573.4-11,289.4)	0.095
IL-18 pg/mL (D0)	5,256.4 (5,003.5-5,728.1)	581.7 (225.0-5,010.6)	**0.012**
MCP-1 pg/mL (D0)	2,411.3 (649.0-442,488.7)	807.0 (354.4-25,332.8)	0.228

1 Quantitative data expressed as median and interquartile range in parentheses. ^2^ Mann-Whitney test was used for comparisons.

### Cytokine Levels and Their Association with the Presence of Persistent Pain

The clinical characteristics of patients with CHIK were evaluated based on the presence or absence of persistent pain. Most clinical variables and medication use did not differ significantly between the groups. In addition, cytokine levels were analyzed in relation to persistent pain in patients with CHIK. No significant differences in cytokine levels were observed between the groups at any of the analyzed time points.

### Comprehensive Analysis of Clinical Characteristics and Cytokine Profiles in Patients with CHIK in Relation to Comorbidity Status

The clinical characteristics and medication use in patients with CHIK on D0 were compared according to the presence of the previously mentioned comorbidities. Patients with comorbidities were significantly older than those without comorbidities [58.0 ± 13.9 vs. 38.7 ± 13.6 years; p < 0.001]. No significant association was observed between comorbidities and persistent pain (p = 0.650) (Table 2).

**TABLE 2:** Characteristics of patients with chikungunya according to the presence of comorbidities.

Variables	No comorbidities n = 22^1^		With comorbidities n = 18^1^		p-value^2^
	n	%	n	%	
Total	22	100	18	100	-
**Age (years)**	38.7 ± 13.6	-	58.0 ± 13.9	-	**<0,001**
**Persistent Pain**	11	55	10	62.5	0.650
**Medication use**	21	95.5	13	81.3	0.159
NSAIDs	3	13.6	4	25	0.640
Dipirone	17	77.3	6	37.5	0.050
Acetaminophen	5	22.7	5	31.3	0.556
Opioids	2	9.1	0	0	0.615
Pregabalin	1	4.5	0	0	>0.999
Dexamethasone	0	0	1	6.3	0.871

1 Categorical data are reported as absolute counts and percentages. Quantitative data are expressed as the mean ± standard deviation. ^2^ Student’s t-test was used for continuous data, and the chi-square or Fisher’s exact test was used for categorical data.

Overall, no significant differences were observed in the levels of most cytokines between patients with and without comorbidities. However, patients without comorbidities exhibited significantly higher baseline levels of MCP-1 (D0) [1,046.8 (538.5-124,678.7) pg/mL vs. 408.0 (314.3-1,541.8) pg/mL; p = 0.045].

Cytokine levels were evaluated on day D21, during the post-acute phase in patients who were or were not using corticosteroids (prednisone). Of the 32 patients who visited on D21, 10 (31.25%) received corticosteroids. No significant differences in cytokine levels were observed between the groups.

Finally, longitudinal variations in cytokine levels were assessed in patients with CHIK during the follow-up period (D0-D360). Significant differences were observed in the IL-10 and IL-18 levels over time. IL-10 levels showed a sustained increase starting at D21, with significant differences between D0 and D180 and between D0 and D360 (p = 0.027). IL-18 levels significantly increased between D0 and D21 (p = 0.020). IL-2 levels increased significantly; however, no significant pairwise differences were detected after type I error correction (Table 3).

**TABLE 3:** Cytokine levels in patients with chikungunya over the follow-up period.

Cytokines	CHIKV n = 40^1^					**p-value**
	D0 n = 38	D21 n = 30	D90 n = 30	D180 n = 31	D360 n = 24	
**GM-CSF pg/mL**	40,000.0 (20,001.3-40,000.0)	30,000.6 (20,001.3-40,000.0)	33,333.8 (20,001.3-40,000.0)	40,000.0 (20,001.3-40,000.0)	40,000.0 (20,001.3-40,000.0)	>0,999
**IFN-γ pg/mL**	4,348.0 (1,088.0-11,116.9)	10,007.6 (4,328.3-20,000.0)	10,109.4 (10,000.5-20,000.0)	10,012.8 (10,000.7-20,000.0)	10,640.6 (10,002.6-15,554.6)	0.196
**IL-1β pg/mL**	9,695.2 (489.1-23,265.5)	12,501.7 (16.1-25,000.0)	12,501.2 (9.1-25,000.0)	12,755.7 (3,089.3-23,239.5)	16,598.8 (16.6-25,000.0)	0.779
**IL-2 pg/mL**	5,652.5 (5,000.1-10,000.0)	5,000.9 (2,6006.-10,000.0)	5,000.3 (1,126.6-8,012.8)	6,307.2 (5,000.1-10,000.0)	10,000.0 (5,314.1-21,051.7)	0.031
**IL-4 pg/mL**	5,302.9 (5,000.1-10,000.0)	5,495.1 (5,000.2-10,000.0)	6,213.2 (5,000.2-10,000.0)	7,667.1 (5,000.2-10,000.0)	6,569.5 (5,000.6-10,150.3)	0.509
**IL-6 pg/mL**	1,039.0 (11.0-2,377.2)	1,714.0 (1.5-3,084.7)	2,341.2 (1.9-9,164.5)	1,631.9 (1.2-4,455.1)	1,679.9 (2.3-4,770.0)	0.406
**IL-10 pg/mL**	6,774.3 (2,109.5-20,011.4)	20,006.6 (20,001.1-28,758.8)	23,269.5 (20,001.6-33,457.2)	25,217.5 (20,001.9-31,064.3)	23,739.3 (20,005.6-27,263.9)	0.027*
**IL-17A pg/mL**	10,001.5 (3,573.4-11,289.4)	10,007.0 (10,002.0-14,851.2)	11,612.5 (10,001.2-20,000.0)	11,197.4 (10,001.8-20,000.0)	11,620.2 (10,004.9-14,253.2)	0.448
**IL-18 pg/mL**	581.7 (225.0-5,010.6)	5,007.6 (5,003.3-5,493.1)	5,120.5 (5,002.9-5,621.0)	5,005.3 (5,002.8-5,635.0)	5,222.3 (5,005.0-5,511.0)	0.020**
**MCP-1 pg/mL**	807.0 (354.4-25,332.8)	2,345.5 (1,049.6-195,325.4)	136,248.5 (3,111.5-198,634.3)	25,027.9 (2,148.7-196,811.7)	6,881.5 (2,613,3-195,361.5)	0.397

1 Quantitative data are expressed as median and interquartile range (IQR). ^2^ The Friedman test was used to compare the dependent groups. Post hoc pairwise comparisons were performed using Holm correction to control for type I errors. *p < 0.05, among D0, D180, and D360. ***p < 0.05 between D0 and D21. Although 40 patients were initially enrolled in the study, two did not attend the D0 visit for blood sample collection. Over time, participant loss occurred, leading to a reduced number of participants at subsequent follow-up points.

Holm correction was applied only when the Friedman test indicated a statistically significant global difference (p < 0.05) across multiple timepoints. In these cases, post hoc pairwise Wilcoxon signed-rank tests were performed, and Holm correction was applied to adjust for multiple comparisons.

For cytokines, such as IL-2, a significant global p-value in the Friedman test indicated a general tendency for change across the evaluated time points. However, after applying Holm correction to the pairwise comparisons, no individual time-point comparisons remained statistically significant. This reflects a global shift in the distribution over time that was not sufficiently strong in any single pairwise comparison to reach statistical significance after controlling for the multiple tests.

Similar to other prospective cohort studies, participant loss was observed over time. On D0, 38 of the 40 patients provided blood samples. On D21 and D90, 30 participants returned for follow-up, whereas on D180 and D360, 31 and 24 participants provided blood samples, respectively.

**FIGURE 1:**
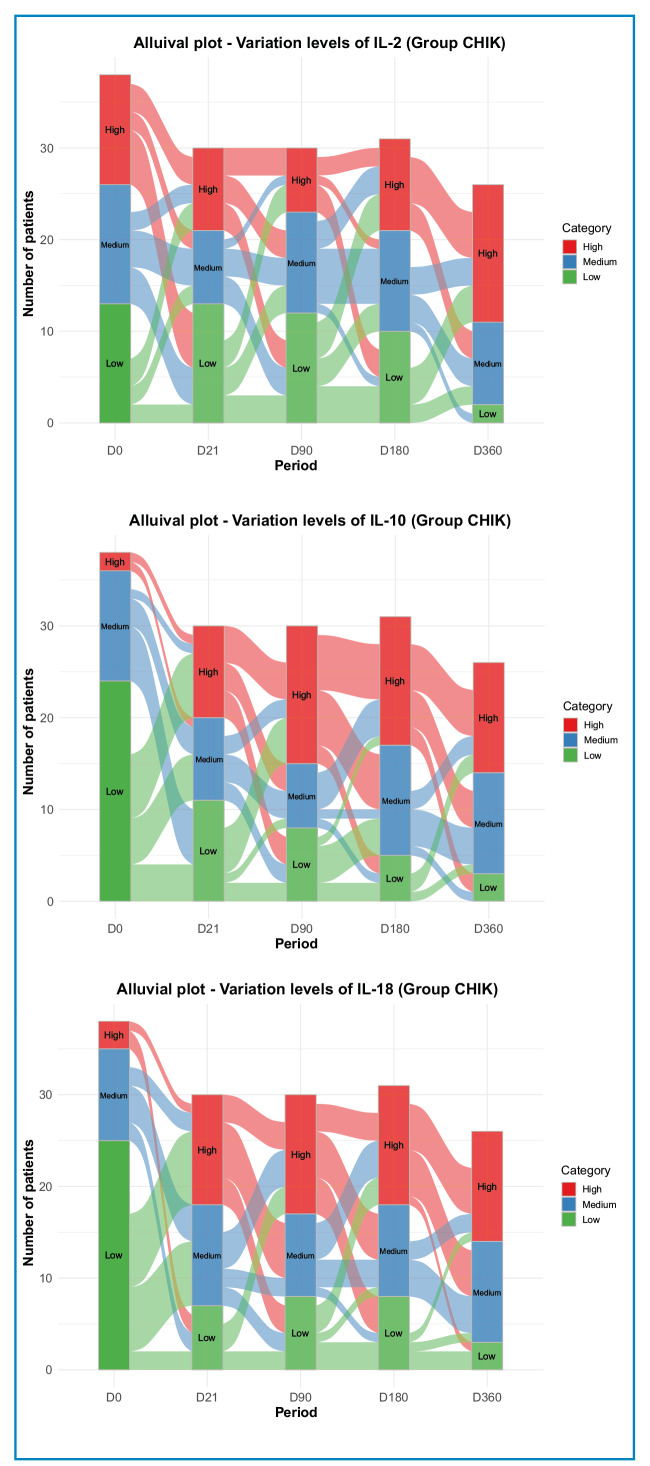
Alluvial plots showing variation in the levels of **(A)** IL-2, **(B)** IL-10, and **(C)** IL-18 in patients with chikungunya throughout the follow-up period.

Alluvial plots were constructed based on cytokine-level tertiles (representing low, medium, and high values) to illustrate the changes in cytokines that showed notable variations over time in patients with CHIK (Figure 1). 

## DISCUSSION

Most female participants in this study (57.9%) experienced persistent joint pain secondary to CHIKV infection. Reports of chronic arthralgia following CHIK infection vary widely in the literature, with prevalence estimates ranging from 14.4% to 87.2%. This variability may be attributed to several factors, including differences in sample size, duration of follow-up, and methods of data collection^22^. In a meta-analysis by Rodriguez-Morales et al., which pooled data from eight studies, the estimated prevalence of chronic CHIK-associated arthropathy in Latin America was 47.57%^23^. Several host-related factors have been associated with a higher risk of developing persistent joint symptoms: age > 40 years, female sex, preexisting joint disease (e.g., osteoarthritis), and prominent joint involvement during the acute phase, particularly in cases presenting with symmetrical polyarthritis and intense inflammatory signs^22^.

In our study, although most patients developed chronic pain, no correlation was observed between the cytokine levels and persistent arthralgia. Possible explanations for this finding include the sample size and patient attrition during follow-up^24^.

The immune system exhibits significant interindividual diversity. The immune responses of different hosts to the same pathogen can vary, as they are the result of gene expression and the influence of several factors, such as age and sex^24^.

Cytokine synthesis is influenced by genetic factors that can be transmitted to the descendants. In our study, the mean age of the participants was 47.3 years, with a range of ± 16.4 years. Age-related immunological diversity due to age may result from immunosenescence. With increasing age, immune-related mutations accumulate, antiviral responses are impaired, and hematopoietic stem cell activity^24^.

Several immune-related genes are located on the X chromosome. Therefore, the differential allocation of various allosomes may explain the differences in immune responses between men and women. Sex hormones influence immune responses. This was observed in response to the influenza vaccine, where a positive correlation was observed between protective antibody levels and estradiol concentrations in women. Other factors that contribute to immunological variations include diet, environmental exposure (such as pollution and chemicals), and the microbiome^24^.

IL-18 is typically elevated during the acute phase of CHIKV infection (16,17), where it promotes IFN-γ release, contributing to viral clearance (18). On day 0 (D0), patients with CHIK in our study exhibited significantly lower IL-18 levels than controls. Dinarello revisited the role of IL-18 in autoimmune diseases and the activity of its natural regulator, IL-18 binding protein (IL-18BP). IL-18BP is constitutively secreted, and by binding to IL-18 with high affinity, IL-18BP negatively regulates its activity of this cytokine^25^ ^,^ ^26^.

In a study by Chirathaworn et al., serum levels of IL-18 and IL-18BP were assessed in patients with CHIK and controls during the acute (samples were collected 2-6 days after the onset of fever) and convalescent (5-13 days after acute-phase sampling) phases. They observed higher IL-18 levels in convalescent phase sera than in acute phase sera, whereas IL-18BP showed the opposite trend and was more abundant during the acute phase. These findings suggest that CHIKV infection induces the IL-18 and IL-18BP expression, with IL-18BP likely acting in the acute phase to modulate IL-18 activity^27^. 

Although IL-18 is classically described as an IFN-γ-inducing cytokine, recent evidence has demonstrated its pleiotropic effects. IL-18 can stimulate T helper cells to produce IL-3, IL-9, and IL-13 and immunoregulate Th2 and Th17 responses^9^. This dual behavior may explain the elevated concentrations of this cytokine observed in our study during the post-acute and chronic phases, potentially reflecting a pro-inflammatory stimulus and an immunoregulatory response.

In a study conducted by Venugopalan et al., who evaluated Indian patients with CHIK, peak levels of Th1 cytokines, such as MCP-1, were observed during the acute phase^16^. Similar results were observed in this study, in which MCP-1 levels were elevated in patients without comorbidities at D0. This may be explained by its role in cell recruitment, which begins in the early stages of the immune response^9^. In a study by Silva et al., MCP-1 concentrations produced by CHIKV-infected monocytes rapidly increased during the acute phase, with serum levels coinciding with the viremic period^28^.

In contrast, in a study by Ninla-Aesong et al., who evaluated cytokine concentrations during chronic phase of CHIK, elevated MCP-1 levels were observed in chronic CHIK. This chemoattractant is expressed at CHIKV infection sites and promotes the migration of monocytes, memory T cells, and NK cells, contributing to persistent joint damage^8^.

IL-10 is a pleiotropic cytokine classically described for its immunoregulatory function. This molecule is primarily produced by Th2 cells, but is synthesized by Th1, Th17, regulatory T cells (Tregs), dendritic cells, and macrophages. Although identified as an anti-inflammatory cytokine, by inhibiting the synthesis of Th1 cytokines, IL-10 can exhibit immunostimulatory effects. This molecule stimulates B cells to differentiate into antibody-secreting plasma cells. Furthermore, IL-10 promotes the proliferation and migration of natural killer (NK) cells and the cytotoxic activity of CD8+ T cells by increasing the production of IFN- γ and granzyme, which optimizes Major Histocompatibility Complex **(**MHC) expression and facilitates antigen recognition^29^.

Several studies have evaluated the dual roles of IL-10. In acute infections, this cytokine modulates the immune response and mitigates the effects of exacerbated inflammation. Conversely, elevated IL-10 levels in acute diseases can suppress the host immune response, favoring pathogen persistence and resulting in chronic infections. Studies related to chronic viral infections, such as cytomegalovirus infections, have shown that blocking IL-10 favors the antiviral activity of T cells, leading to viral eradication. Chronic autoimmune diseases such as rheumatoid arthritis are associated with increased IL-10 levels^29^.

In our study, IL-10 levels were reduced in patients with CHIK in the acute phase and significantly increased during the chronic phase. IL-10 was measured in a study by Venugopalan et al., who reported peak concentrations in patients with CHIK and prolonged symptoms (15-30 days after infection)^16^.

Studies on post-coronavirus disease (COVID) manifestations, such as chronic joint pain, have described an inverse correlation between serum IL-10 levels and pain intensity. This finding suggests that this cytokine has an analgesic function in persistent musculoskeletal pain^29^.

Kulkarni et al. investigated Treg cell activity and IL-10 concentrations in patients with CHIK. Tregs play a modulatory role in the regulation of CD4+ and CD8+ T cell activity. These regulatory effects may result from cytokine secretion, such as IL-10. The cited study showed no significant differences in IL-10 levels among patients in the acute, chronic, and control groups; however, IL-10 concentrations were higher in recovered patients, highlighting the anti-inflammatory potential of this cytokine^30^.

Therefore, in the current study, elevated IL-10 levels in the chronic phase of CHIK may represent immunomodulatory and pro-inflammatory mechanisms.

Chronic CHIK-associated arthropathy may result from multiple underlying mechanisms, including the persistence of viral genetic material with continued CHIKV replication, retention of viral antigens that are not adequately cleared, sustained antigenic stimulation and inflammation, or immune-mediated inflammatory responses that persist even after viral elimination^31^.

Several studies have investigated cytokine concentrations in patients during the chronic phase of CHIKV infection. Ninla-Aesong et al. reported increased levels of IL-1β, IL-6, and IL-8 in individuals with chronic symptoms compared to controls^8^. Chow et al. observed elevated IL-17 levels at this stage, whereas Chaaithanya et al. reported increased IL-6, IL-8, and MCP-1 levels. These cytokines are known to contribute to the pathophysiology of other inflammatory arthritis, including rheumatoid arthritis and Ross River virus-associated disease, suggesting a potential overlap in immunopathogenic mechanisms with chronic CHIK arthritis^32^ ^,^ ^33^.

In this study, IL-18 and IL-10 levels remained persistently elevated during the chronic phase, which has not been previously reported. Sustained IL-18 and IL-10 expression may reflect ongoing pro-inflammatory and regulatory immune activities. The anti-inflammatory response aims to limit long-term immune-mediated tissue damage secondary to CHIKV infection.

The immune response against other arboviruses involves cytokines. Dengue virus infection activates endothelium and recruits several cells. Endothelial cells release IL-6, IL-8, and TNF-alpha, which contribute to thrombocytopenia, bleeding, and hepatitis. Another cytokine produced is IL-6, which may be associated with severe dengue manifestations^34^. Type I IFN (IFN-Is) are produced by myeloid and epithelial cells in response to Zika virus (ZIKV) infection. This molecule is important for antiviral defense. By evading the action of IFN-I, ZIKV crosses the placenta during pregnancy and causes neurological damage in the fetus. In addition to these cytokines, GM-CSF, IL-1B, IL-2, IL-6, IL-17, and IL-22 have been observed in the acute phase of Zika virus infection. In the late phase of Zika, IL-1B and IL-2 continue to be produced, which does not occur in late dengue infection. In both arboviruses, elevated IL-10 concentrations were recorded in the late phase, similar to the results of this study^35^.

A comparative analysis of our findings with those of other studies revealed notable variations in the cytokine profiles across the different phases of CHIKV infection. These discrepancies may be attributed primarily to host genetic variability, including polymorphisms in genes encoding cytokines, transmembrane proteins, enzymes, cellular mediators, receptors, and pattern-recognition molecules. Such genetic differences can significantly modulate the magnitude and quality of immune responses against CHIKV infection^9^ ^,^ ^36^. Other potential contributors to the observed differences include variations in sample size, timing of sample collection, disease phase at the time of sampling, circulating CHIKV genotypes, analytical methods used in cytokine quantification, and interindividual variability in cytokine expression during the course of CHIK^16^ ^,^ ^18^ ^,^ ^37^. 

This study has several limitations. This study included a small number of patient and control samples. Attrition over time reduced the sample size in the chronic phase (the study was initiated with 40 patients and concluded with 24 patients), which may have influenced the results of this phase. Furthermore, the inclusion of only female participants precluded comparisons with male participants. 

Nevertheless, it is important to highlight that this is the first Brazilian study and one of the few worldwide studies to longitudinally follow patients with CHIK over a 12-month period. It included systematic assessments of persistent joint pain and cytokine profiling at five distinct time points during the illness.

## CONCLUSION

In this study, more than half (57.9%) of the women diagnosed with CHIKV infection reported persistent arthralgia. Nonetheless, no statistically significant differences in cytokine levels were observed between patients with or without comorbidities or between those with ongoing joint symptoms and those who had fully recovered. IL-18 levels were lower in patients with CHIK than those in controls at baseline (D0), followed by an increase from the post-acute phase onwards, which persisted through the chronic phase. IL-10 levels were reduced on D0 in patients with CHIK, with an increase by D21, and a more pronounced and sustained elevation during the chronic phase. This dynamic cytokine behavior throughout the CHIK phase has been observed in other studies and may be influenced by factors, such as host immunogenetics. Further research on cytokine kinetics in CHIK, such as IL-10 and IL-18, is essential to better understand the disease pathophysiology and explore their potential as therapeutic targets and biomarkers of chronicity and disease severity.

## Data Availability

Research data is available upon request.
